# Transcriptome Analysis of *Lolium temulentum* Exposed to a Combination of Drought and Heat Stress

**DOI:** 10.3390/plants10112247

**Published:** 2021-10-21

**Authors:** Ruth C. Martin, Brent A. Kronmiller, James E. Dombrowski

**Affiliations:** 1 USDA-ARS, National Forage Seed Production Research Center, 3450 SW Campus Way, Corvallis, OR 97331-7102, USA; dombrowskijim2020@gmail.com; 2 Center for Quantitative Life Sciences, Department of Botany and Plant Pathology, Oregon State University, Corvallis, OR 97331-7102, USA; Brent.Kronmiller@cgrb.oregonstate.edu

**Keywords:** abiotic stress, drought stress, heat stress, hormones, *Lolium temulentum*, RNA-sequencing, transcription factors

## Abstract

Drought and heat are two major stresses predicted to increase in the future due to climate change. Plants exposed to multiple stressors elicit unique responses from those observed under individual stresses. A comparative transcriptome analysis of *Lolium temulentum* exposed to drought plus heat and non-stressed control plants revealed 20,221 unique up-regulated and 17,034 unique down-regulated differentially regulated transcripts. Gene ontology analysis revealed a strong emphasis on transcriptional regulation, protein folding, cell cycle/parts, organelles, binding, transport, signaling, oxidoreductase, and antioxidant activity. Differentially expressed genes (DEGs) encoding for transcriptional control proteins such as basic leucine zipper, APETALA2/Ethylene Responsive Factor, NAC, and WRKY transcription factors, and Zinc Finger (CCCH type and others) proteins were more often up-regulated, while DEGs encoding Basic Helix-Loop-Helix, MYB and GATA transcription factors, and C2H2 type Zinc Finger proteins were more often down-regulated. The DEGs encoding heat shock transcription factors were only up-regulated. Of the hormones, auxin-related DEGs were the most prevalent, encoding for auxin response factors, binding proteins, and efflux/influx carriers. Gibberellin-, cytokinin- and ABA-related DEGs were also prevalent, with fewer DEGs related to jasmonates and brassinosteroids. Knowledge of genes/pathways that grasses use to respond to the combination of heat/drought will be useful in developing multi-stress resistant grasses.

## 1. Introduction

Forage and turf grasses are exposed to many biotic and abiotic stresses that impact yields and the quality of forage, seed yield, and turfgrass utility. Drought and heat are two major stressors predicted to increase in the future due to the changing climate [1]. In the US, there have been nine drought related disasters since 2010, four of which were concurrent with major heat events. It was estimated that these natural drought and combined heat and drought (heat/drought) disasters caused USD 85 billion in agricultural losses according to data from the NOAA National Centers for Environmental Information (NCEI) U.S. Billion-Dollar Weather and Climate Disasters [2]. With the increasing likelihood of these events in the future, it is important to understand how the grasses respond to these combined heat/drought disasters to facilitate the development or identification of crops that can perform better under these increasingly extreme conditions. 

Heat stress negatively impacts many aspects of crop production including germination, biomass accumulation, and floral and seed development, all of which can affect forage and seed yields. Excessively high temperature affects many physiological processes in plants leading to reduced photosynthesis, altered water and nutrient uptake, and increased evapotranspiration. At the cellular level, damage to proteins, membranes, mitochondria, photosynthetic machinery, and chloroplasts, and increases in reactive oxygen species (ROS) are common during heat stress. The photosystem reaction centers are impacted, with PSII being more sensitive to heat stress. There is a loss of chlorophyll pigments due to lipid peroxidation during heat stress [3]. As part of the heat shock response, plants produce heat shock proteins that act as chaperones to protect proteins from aggregation and assist in the folding or unfolding of proteins to achieve proper conformation. Plants also utilize a complex network of signaling molecules, hormones, and transcription factors to modulate changes in gene expression in response to heat stress. High temperatures can greatly reduce the performance of forage and turfgrass species. Perennial ryegrass (*Lolium perenne* L.) is an important cool season grass species that is utilized for forage and turf worldwide. The optimal temperature range for growing perennial ryegrass is between 16 and 24 °C. A recent study examined the transcriptional response of perennial ryegrass in response to heat stress [4]. They identified up- and/or down-regulated transcripts that encode heat shock proteins (HSPs), signal transduction factors, and transcription factors. Many of the HSPs were found to be present only in the heat stressed sample. Transcription factors that were highly differentially regulated included HSF, AP2/ERF, MYB, bHLH, and Divaricata. Similar to other species, genes encoding components of photosystem II were differentially regulated under heat stress in perennial ryegrass [4]. Enriched GO categories included ‘response to abiotic stimulus’ and ‘antioxidant activity’. Within the antioxidant response genes, many of the superoxide dismutase encoding genes were up-regulated. Most peroxidase encoding genes were down-regulated, except the ascorbate peroxidase genes, which were mainly up-regulated. Catalase genes were up-regulated. This suggests that oxidative stress is an important component of the heat stress response in perennial ryegrasses. 

Drought stress affects multiple phases during crop production, including seed germination, stand development, plant growth, tillering, and floral and seed development, each of which may lead to reduced productivity. Plants respond to drought by closing their stomata and reducing leaf growth, and some plants may respond by increasing root growth, inducing senescence, and abscising leaves. During drought stress in Arabidopsis, a small signaling peptide, CLAVATA3/EMBRYO-SURROUNDING REGION-RELATED 25 (CLE25), has been shown to transmit a signal from drought-stressed roots to the leaves, where it induces ABA biosynthesis leading to abscisic acid- (ABA) regulated stomatal closure and control of transpiration in leaves [5]. Other small signaling molecules such as ABA, phytohormones, calcium, and proteins such as the mitogen-activated protein kinases (MAPK) and phosphatases are utilized to transmit signals to effect transcriptomic changes in response to drought [6]. These transcriptional changes result in the accumulation of compatible solutes, such as proline and late embryogenesis abundant (LEA) proteins, antioxidants, and ROS-scavenging enzymes that help to maintain leaf water potential and protect cellular components from ROS-induced damage [7,8]. Extensive signaling networks involving a variety of small signaling molecules, hormones, and transcription factors are utilized to mediate the response to adapt and protect the plant against drought stress. 

The ability to maintain turf quality and growth when exposed to drought stress varies widely in perennial ryegrasses. In a comparison of drought sensitive and drought tolerant accessions, leaf wilting and decreases in relative water content were much greater in the sensitive accession [9]. Analysis of differentially expressed genes between the drought sensitive and drought tolerant accessions was used to identify genes that may be important in adapting to drought stress conditions [9]. They identified several genes that were more prevalent in the drought tolerant genotype that encode for proteins involved in signal transduction (MAPK2) and proteins important for detoxifying ROS, such as Cu/Zn SOD and glutathione peroxidase. A gene encoding dehydrin was found to be up-regulated in both accessions in response to drought stress. Other genes encoding for proteins more prevalent in the drought sensitive accession included HSPs and trehalose synthesis enzymes, which act to help maintain membrane fluidity and stabilize proteins under drought stress [9]. Overall, the genes differentially expressed between the two accessions encoded proteins involved in amino acid, lipid and carbohydrate metabolism, signal transduction, transcription, photosynthesis, protein synthesis, detoxification, and energy [9]. These genes are likely important for the adaptation of perennial ryegrasses to drought stress.

Many studies have looked at responses to drought or high temperature, but plants in the field often are exposed to heat/drought stress simultaneously. Early studies comparing responses to heat or drought stresses versus combined heat/drought stresses in tobacco revealed that several genes down-regulated in response to combined heat/drought stress were actually induced in plants exposed to single drought or heat stresses [10]. Differentially expressed genes (DEGs) that were unique to the combined heat/drought stress were also discovered [10]. Studies in Arabidopsis revealed that exposure to heat and drought together elicits a transcriptomic response with shared and unique DEGs to those observed in response to individual stressors [11]. Approximately 40% of the up- and down-regulated DEGs identified in the combined heat/drought stressed plants were unique from those observed in response to the individual stresses [11]. The combination of heat/drought stress in different species often resulted in a greater suppression of photosynthesis, a reduced functioning in photosystem II, and increased leaf temperatures and ROS than that found when exposed to individual heat or drought stress [12]. With the likely increase in the occurrence of combined heat/drought events in the future, a better understanding of how grasses respond to the combined heat/drought stresses is necessary to facilitate the development of crops better able to adapt to these stresses. 

Grass species are generally polymorphic, obligate out-crossing species that are self-incompatible, which results in considerable genetic diversity between individual plants of the same variety. This inherent heterogeneity between individual plants can result in inconsistent or undesirable variation between plants that introduces another layer of complexity to the analysis when investigating the transcriptional response of a plant to stress. In contrast, the model grass species *Lolium temulentum* (*Lt*) is a diploid self-fertile grass species that has inbred lines. Furthermore, it is member of the *Lolium* genus, and it possesses morphological traits similar to common forage and turf grass species [13,14,15]. Due to these characteristics, this model grass species was chosen for this study. 

This paper describes the transcriptomic response of *Lt* to the combined effects of heat/drought stresses, with an emphasis on transcription factors and hormone-related processes. While most transcription factors had both up- and down-regulated DEGs, the heat shock transcription factor DEGS were all up-regulated. Hormone-related DEGs encoded for proteins related to biosynthesis, transport, degradation and/or inactivation. Auxin-related DEGs were the most prevalent, encoding for auxin response factors, binding proteins, and efflux and influx carriers. Gibberellin-, cytokinin- and ABA-related DEGs were also prevalent, with fewer DEGs related to jasmonates and brassinosteroids. 

## 2. Results and Discussion

### 2.1. Plant Phenotype after Heat/Drought Treatments

**Figure 1:**
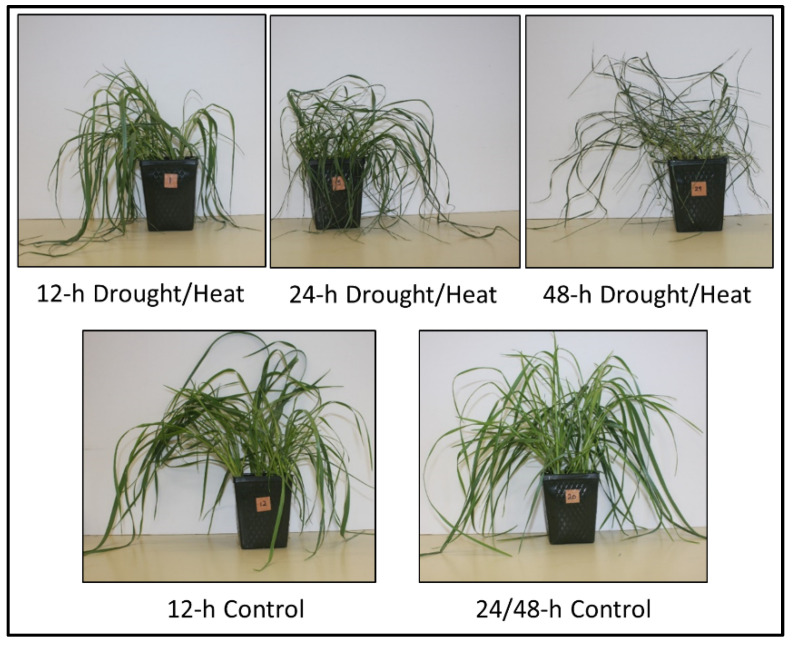
Images of representative *Lolium temulentum* plants after being exposed to heat/drought stress conditions for 12, 24, or 48 h (**top panel**) and control plants at 12 and 24/48 h (**bottom panel**).

**Figure 2:**
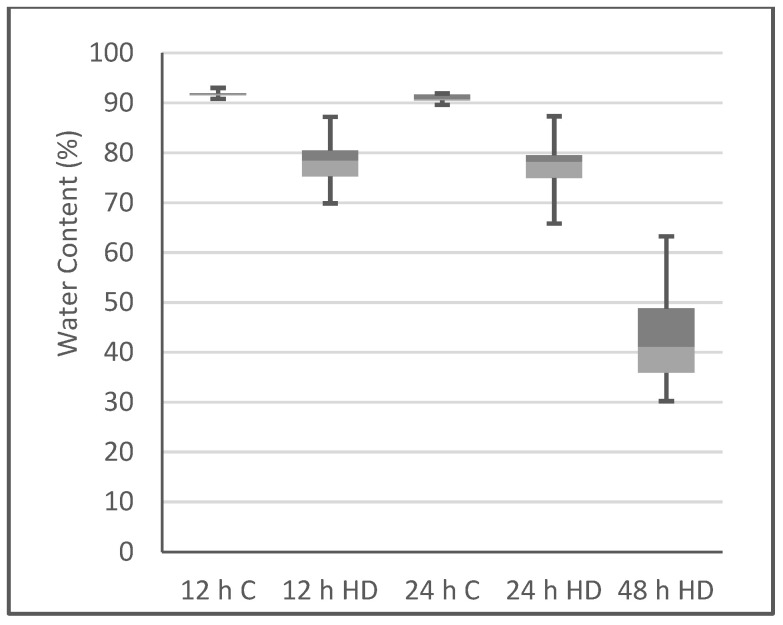
Boxplots of the average percentage of water content ((FW − DW)/FW) × 100) of control (C) and heat/drought treated plants (HD) at 12, 24, and 48 h time points. FW, fresh weight; DW, dry weight.

After plants were exposed to heat/drought for 12 h, wilting of the plants was evident, which was increased further after 24 and 48 h of treatment (Figure 1). Plants treated for up to 48 h were able to be rescued upon watering, but after exposure to heat/drought for 72 h the plants could not be rescued; therefore, these plants were not included in the experiment. The water content of the control and heat/drought stressed plants was determined at each time point (Figure 2). After 12 and 24 h of stress, the water content was still quite high, at around 78%, but by the end of 48 h, the water content decreased to about 40%.

### 2.2. RNA-Seq Libraries

RNA sequencing was generated from heat/drought treated plants and control plants at three time points. Sequencing gave an average of 12.7 M paired end reads per sample (minimum 10.4 M, maximum 15.0 M). Total sequencing reads and rate of alignment to the *Lt* transcriptome are shown in Table 1.

**Figure 3:**
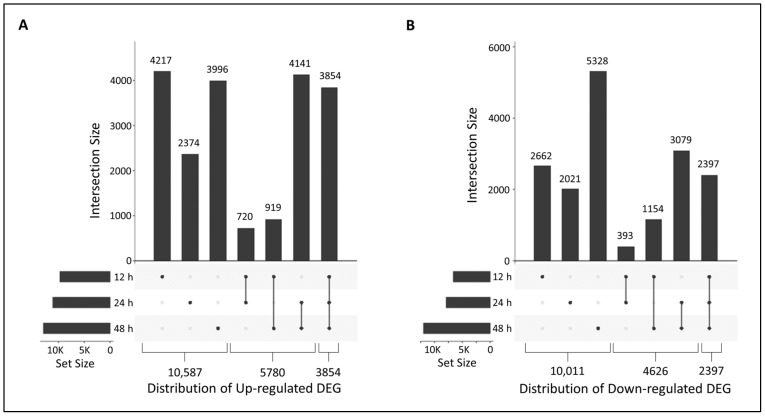
UpSetR plots depicting the number of unique and shared differentially expressed genes (DEGs) between control and heat/drought stressed plants at each time point. Graph depicts the number of unique, total, and shared DEGs that were (**A**) up-regulated and (**B**) down-regulated. The number above each bar represents the number of DEGs in each category. Shared categories are indicated by linked dots below the x-axis. The numbers below the chart represent the total number of DEGs present at only one, two, or all three time-points. Data sets are presented in Supplementary Tables S1 and S2.

The *Lt* reference transcriptome developed previously for other stress-associated transcriptome studies was used in this study [16,17]. The transcriptome was constructed using RNA-Seq from control and green leaf volatile-treated plants (plus some older 454 RNA-Seq data from salt-treated plants and control plants), and therefore may have been missing highly specialized up-regulated genes only seen in heat/drought stress responses. Differentially expressed sequences were determined by comparing fragments per kilobase million (FPKM) values for transcripts of the drought/heat treated plants and the control plants from the same time points. Values were based on false discovery rates and *p*-values ≤ 0.01 and |log_2_—fold changes| ≥ 1. Overall, there were 33,709 up-regulated DEGs and 26,454 down-regulated DEGs in the combined 12, 24, and 48 h data sets, with 20,221 unique up-regulated and 17,034 unique down-regulated DEGs. The distribution of the unique and shared up- and down-regulated DEGs at the various time points are presented in the UpsetR plots shown in Figure 3A,B, respectively. As indicated in the set sizes for each time point depicted on the left axis, the total number of DEGs increased at increasing stress exposure times with 9710, 11,089, and 12,910 up-regulated and 6606, 7890, and 11,958 down-regulated DEGs at 12, 24, and 48 h, respectively. The data (id, fold change, annotations, and sequences) for up- and down-regulated DEGs for each stress time point and the DEGs shared between time points are presented in Supplementary Tables S1 and S2, respectively. In terms of unique genes, the 12 h time point had the most unique up-regulated DEGs, the 48 h time point had the most unique down-regulated DEGs, while the 24 h time point had the fewest unique up- and down-regulated DEGs for all time points. The 12 and 24 h stressed samples had the fewest DEGs in common for both the up- and down-regulated DEGs, followed by the 12 and 48 h stressed samples. The 24 and 48 h time points shared the most DEGs for both the up- and down-regulated categories. While this was probably due to the increased exposure to heat/drought stress at these later time points, it may also have been affected by interactions between the circadian clock and stress responses in plants [18]. The 12 h time point was taken at the end of the light period, while the 24 and 48 h time points were taken at the beginning of the day, after the plant was exposed to cooler nighttime temperatures. There were also many up- (3854) and down-regulated DEGs (2397) that were shared among all three time points. Approximately 17% of the up-regulated and 8% of the down-regulated DEGs were lacking annotations. It would be interesting to see if the DEGs lacking annotations in our study were also found in heat/drought studies in other species. These unannotated common DEGs would be of interest for further analysis. 

### 2.3. Gene Ontology Analyses

The genes differentially regulated during heat/drought stress at the different time points were investigated further using the WEGO (Web Gene Ontology Annotation Plot) program. Graphs showing the forty most significant GO categories and their p-values for Chi-square tests at the 12, 24, and 48 h time points are shown in Supplementary Figure S1A–C. The most significantly differentially regulated categories present at all time points included protein folding, transcriptional regulation, cell (cell cycle cell division, cell part, plasma membrane, actin and tubulin), organelles (mitochondrial membrane parts and protein complex, endoplasmic reticulum membrane, and ribosomes), binding (ion, small molecule, carbohydrate derivative, drug, protein), transport, signal receptors, molecular transduction, oxidoreductase and antioxidant activity. Many of these categories represent components of and/or processes related to sensing, transducing, and responding to abiotic stresses. Categories highly significant at the 24 and 48 h time-points related to catalytic activity (protein, RNA, and DNA), apoplast, and secondary metabolic processes may play an important role in regulating transcription, ROS, and hormones in response to stressors. 

**Figure 4:**
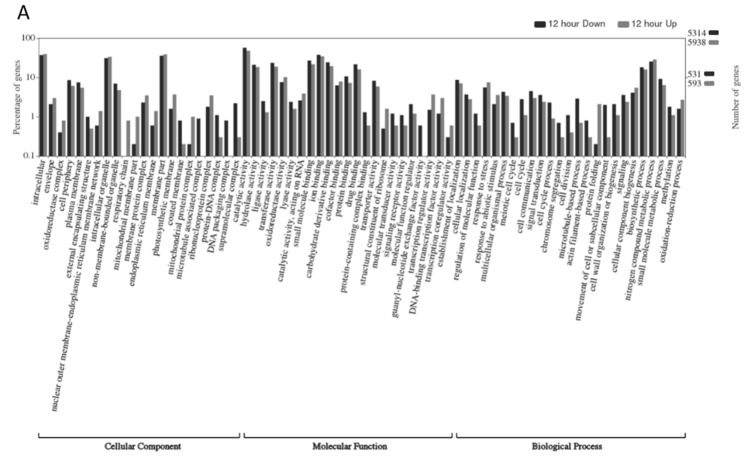

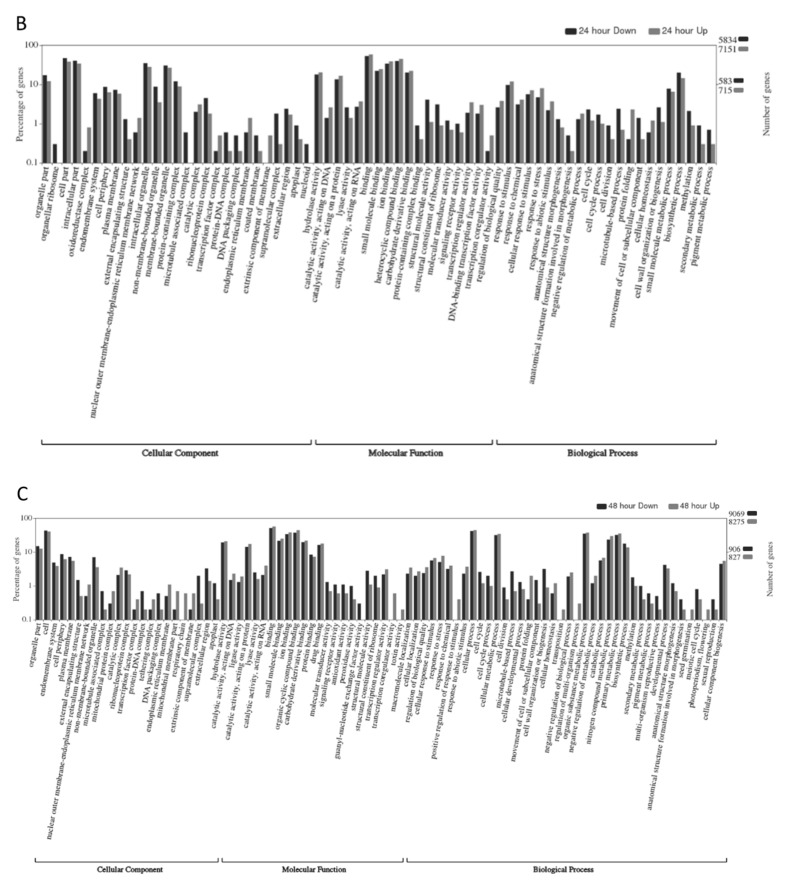
Gene ontology analysis for DEGs up- and down-regulated in response to heat/drought stress after (**A**) 12 h exposure, (**B**) 24 h exposure, and (**C**) 48 h exposure. Black bars = down-regulated DEGs; Gray bars = up-regulated DEGs. Numbers on the left-hand axis represent the percentage of genes (displayed on a log_10_ scale). Numbers on the right axis represent the number of total DEGs (top numbers) and 10% of the DEGs (middle numbers) contained in up-regulated (gray) and the down-regulated (black) datasets used in analysis.

To further examine their potential role in the drought/heat response, WEGO plots were produced for each time point to visualize the number of DEGs (FDR and *p*-values < 0.01, and |Log_2_ FC| ≥ 1) that were up- or down-regulated in each category (Figure 4A–C; 12, 24, 48 h of stress, respectively). The WEGO plots are divided into three categories: cellular component, molecular function, and biological processes. An overall examination of the WEGO plots reveals many categories with similar levels of up- and down-regulated DEGs. Categories that had a greater proportion of up-regulated DEGs at all time points included: cellular component (nuclear outer membrane-endoplasmic reticulum membrane network, ER membrane); molecular function (transcriptional regulator or coregulatory activity); and biological process (protein folding, and response to stress or stimulus). Categories with a greater proportion of down-regulated DEGs at all sampling points included: cellular component (cell periphery, external encapsulating structure, non-membrane-bounded organelle, microtubule associated complex, protein–DNA complex, DNA packaging complex and supramolecular complex); molecular function (lyase activity, molecular transducer activity, signaling receptor activity); and biological processes (cell cycle, cell division, cell wall organization or biogenesis, movement of cell or subcellular component, microtubule-based process and methylation). Our analysis found some categories with a greater proportion of up-regulated DEGs that were common between the 12 and 48 h sampling times included respiratory chain and mitochondrial membrane parts. Similarly, down-regulated DEGs shared between the 12 h and 48 h time points included protein binding, guanyl-nucleotide exchange factor activity, and meiotic cell cycle. Interestingly these categories were not as prominent at the 24 h time point. This could be due to the plants being fully stressed at 12 h, but they may have been able to partially recover during the night cycle. However, at 48 h, the plants may not have been able to recover as fully after two full days of stress. 

### 2.4. Analysis of Transcription Factors Differentially Regulated during Heat/Drought Stress

**Figure 5:**
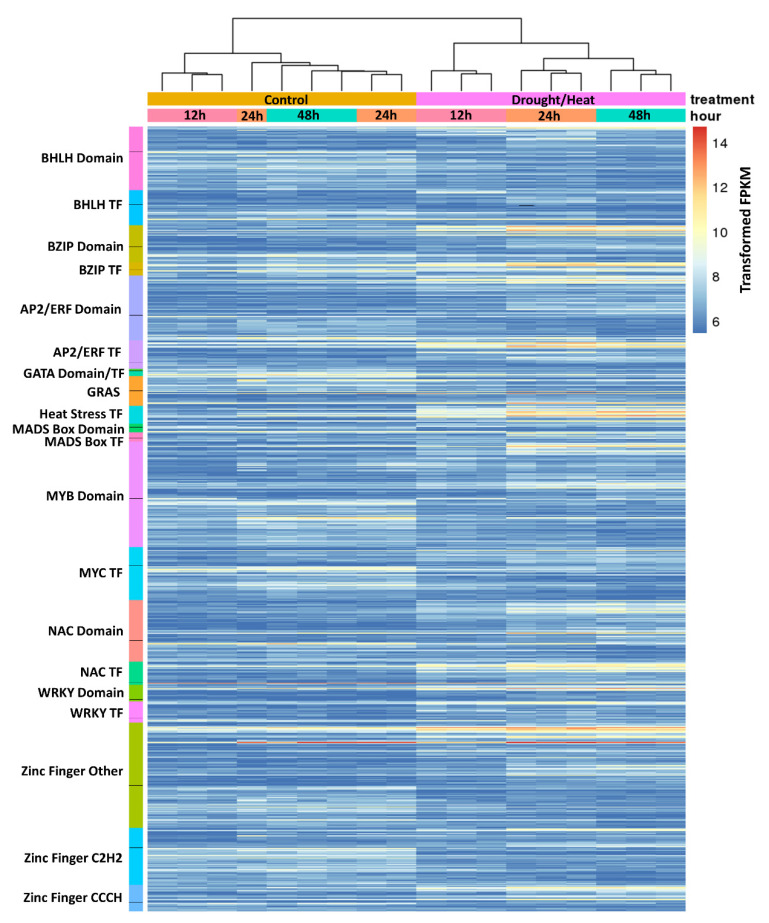
Heatmap of the 914 DEGs that are annotated as transcription factors or domain containing proteins and that also passed the differential expression filter of *p*-value 0.01, FDR 0.01, and log_2_-fold changes less than − 2 or greater than 2 in at least one time point. Values were transformed with variance stabilizing transformation. Gradient bar on the right side of the heat map represents the transformed value of fragments per kilobase per million reads (FPKM). Treatments (control and heat/drought) and treatment times of 12, 24, and 48 h are as shown. Gene groups of transcription factor DEGs are shown to the left of the rows. Lines in each transcription factor class on the left axis separate the up- and down-regulated DEGs in each class. Samples (columns) are clustered using Euclidean distance and a dendrogram is shown above the heatmap. For the specific expression data for each individual transcription factor represented in the heatmap, see Supplementary Table S3.

Transcription factors play an important role in regulating gene expression during normal growth and development and in response to abiotic and biotic stresses. Up- and down-regulated DEGs encoding transcription factors were further examined to determine their potential involvement in the heat/drought response in *Lt*. A heat map showing the expression levels (FPKM) of transcription factors that were differentially expressed by |log_2_ fold| ≥ 2 between control and heat/drought treated plants in at least one time point is shown in Figure 5. Expression data for transcription factors included in the heatmap are presented in Supplementary Table S3. The heat map shows a clear separation between the 12 h control samples and the 24 and 48 h control samples. This may be due to the circadian cycle; the 12 h samples were taken at the end of the light-cycle, while the 24 and 48 h samples were taken at the beginning of the light cycle. Similarly, there is only a small difference between the 24 and 48 h control samples as they represent plants in the same light-cycle that only differed in age by one day. In contrast, the stressed samples were grouped into their respective time points, as expected. The heat map includes transcription factors as well as DEGs that contain specific transcription factor domains in that category; for example, NACs (NAM, ATAF1/2, CUC2) include up- and down-regulated NAC-domain-containing DEGs followed by up- and down-regulated NAC transcription factor DEGs. This provides a global representation of the dynamic changes occurring in transcriptional regulation induced by heat drought stress within the plant.

The BZIP (basic leucine zipper), AP2/ERF (APETALA2/Ethylene Responsive Binding Factor), heat stress, NAC, WRKY transcription factors, and Zinc Finger (CCCH type and others) proteins displayed more up-regulated DEGs, while the BHLH (basic Helix-Loop-Helix), MYB (myeloblastosis), and GATA transcription factors, and C2H2 type Zinc Finger proteins were more often down-regulated. However, it should be noted that the up- or down- regulation of transcription factors may not lead to a corresponding increase or decrease in the expression of genes regulated by those specific transcription factors.

The DEGs annotated as zinc finger proteins, which were divided into three categories (C2H2 zinc fingers, CCCH zinc fingers, and other zinc fingers), were the most abundant DEGs in the heat map. In addition to their role in plant growth and development, many of the C2H2 zinc finger proteins are thought to be an integral part of networks that sense and respond to various types of biotic and abiotic stress. They can bind DNA, RNA, and proteins to induce or repress gene expression and have been shown to play a role in osmotic adjustment, ROS scavenging, and hormone signal transduction [19,20]. The DEGs annotated as C2H2 type zinc finger proteins were more often down-regulated and present at the later time points, while the up-regulated C2H2 zinc finger DEGs were more prevalent at 12 h. While there were fewer C3H zinc finger DEGs overall, they were more prevalent in the up-regulated DEGs. The C3H zinc finger proteins are thought to bind to RNA and have been shown to be involved in flower development, in modulating salt stress responses, responding to drought and ABA, and affecting cold stress tolerance [21,22,23,24,25,26]. The zinc finger DEG category encoded for other types of zinc finger containing proteins including RING-Finger proteins, A20/AN1 stress associated proteins (SAPs), Zf-HD dimerization-type, zinc finger primase proteins, and zinc finger BED proteins. The RING-Finger DEGs were more often up-regulated and were distributed evenly between all time points, whereas the down-regulated RING-Finger DEGs were more prevalent at the later time points. These proteins are involved in protein–protein interactions and some act as E3 ubiquitin ligases, an important component of the ubiquitin proteasome system, which plays an important role in hormone signaling and abiotic stress responses [27,28,29]. There were also a few zinc finger BED proteins in both the up- and down-regulated DEGs. Some zinc finger BED proteins, when overexpressed in tobacco or rice, positively regulate stress-responsive genes and have been shown to increase drought resistance in rice [30]. The *Drosophila* DREF and human ZBED1, both zinc finger BED proteins, have been found to play a critical role in the regulation of genes involved in cell proliferation, DNA replication, chromatin structure, apoptosis and differentiation [31]. It is possible that zinc finger BED proteins could provide similar functions during stress responses in plants.

Another category of zinc finger proteins with a very high number of DEGs, but not included in the heat map, are the ZF-rvt (reverse transcriptase) domain-containing proteins. Some ZF-rvt domain-containing genes are thought to be derived from non-LTR retrotransposons that were endogenized in plants during evolution and have been proposed to modulate the expression of adjacent genes [32]. These were much more prevalent in the up-regulated DEGs and at the later stages of drought and heat stress, while the down-regulated ZF-rvt DEGs were less prevalent overall and peaked at the 24 h time point.

Members of the MYB TFs have been shown to be involved in controlling plant development, metabolism, cell cycle, cell wall biosynthesis, cell fate, and abiotic and biotic stress responses [33]. They also are involved in stomatal closure and responses to ABA, drought, salinity, and cold temperatures [34]. In Arabidopsis, the MYB30 transcription factor is involved in heat and oxidative stress responses through calcium signaling [35]. When *MYB12* and *MYB75* were overexpressed in Arabidopsis, plants overaccumulated anthocyanins, which were shown to control ROS, leading to enhanced oxidative and drought stress tolerance [36]. In the current study, the MYB protein family had a large number of DEGs. The down-regulated MYB domain-containing protein DEGs were present at the later time points and were slightly more prevalent than the up-regulated DEGs, which were more abundant at the 48 h time point. The down-regulated MYB TF DEGs were much more prevalent than the up-regulated MYB TF DEGs, and both were more abundant at the later time points. The MYB TFs are thought to interact with the basic helix-loop-helix (bHLH) TFs in the regulation of various processes including cold-stress tolerance, phytochrome signaling, and flavonoid biosynthesis [37,38,39]. Members of the bHLH family of transcription factors are involved in fruit and stomatal development, light signaling, seed germination, responses to drought, salt, low temperature stress, and ABA homeostasis [40,41]. In maize overexpressing the bHLH transcription factor, *ZmPTF*_1_, an increase in ABA, ABA signaling, and the up-regulation of several stress-responsive transcription factors including AP2/DREBP, WRKY, NAC and bHLH was observed [42]. The bHLH protein, OsbHLH148, was shown to interact with proteins in the jasmonate signaling pathway and to confer drought tolerance when *OsbHLH148* was overexpressed in rice [43]. In our study, the bHLH protein DEGs were more often down-regulated and present at the later time points, and the up-regulated DEGs were more frequent at the 24-h time point.

The bZIP (basic region/leucine zipper motif) class of TFs have been demonstrated to play a role in normal plant development (seed, floral, leaf, and vascular development), and responses to abiotic and biotic stresses [44]. A large number of bZIP TFs have been shown to be responsive to drought and/or heat stress in rice. When overexpressed in rice, many of these TFs conferred enhanced drought tolerance (OsbZIP23, OsbZIP72, OsbZIP16, OsBZIP46, OsbZIP71, OsbZIP66, OsbZIP42, OsbZIP62) [45,46,47,48,49,50,51,52]. Transgenic plants overexpressing *OsBZIP62* are also more tolerant of oxidative stress [52]. Other bZIP transcription factors are involved in ER stress responses [53] and the unfolded protein response that occurs when plants are exposed to stress [53]. In a study of bZIP transcription factors in wheat, Agarwal et al. [54] identified a bZIP transcription factor gene that was responsive to salt, heat, drought, and ABA. They found that this gene was not induced upon heat stress in heat tolerant varieties, and that Arabidopsis overexpressing this bZIP TF gene performed better than control plants under salinity, drought and heat stress conditions, and had a lower accumulation of ROS under heat stress [54]. Another group of bZIP proteins heterodimerize to form the C/S_1_ bZIP network, which is thought to alter the plants metabolism to adapt and survive under low energy conditions often present during abiotic stresses [55]. In this analysis, DEGs encoding bZIP proteins were more often found to be up-regulated in heat/drought stressed plants. The DEGs encoding bZIP domain-containing proteins were more prevalent and equally distributed among the different time points, while the DEGs encoding bZIP TFs were more often observed at the earlier time points. There were only a few down-regulated bZIP TFs DEGs present at the later time points. Overall, these data suggest that the bZIP domain containing proteins and TFs may be a useful target for developing heat/drought tolerant plants.

Members of the NAC (NAM, ATAF1,2, CUC2) proteins and transcription factors have been shown to be responsive to biotic and abiotic stresses in plants and are also involved in ROS detoxification, senescence, the hypersensitive response (OsNAC4), and the DNA damage response [56,57,58]. Interactions between hormone signaling pathways (SA, JA, ACC, ABA, GA, auxins, ERFs) [59], NAC TFs, and other TFs (MYB, bZIP, and DREB/CBF) are important in the NAC-mediated stress responses [56]. Rice overexpressing various NAC TFs (*OsNAC045*, *OsNAC1*, *OsNAC10*) showed improved drought and/or salt tolerance [60]. The *OsNAC3*-overexpressing rice was more tolerant to heat, drought, and oxidative stress [61], while rice overexpressing *OsNAC2* was more tolerant to the cold [62]. In response to drought and heat in this study, the DEGs encoding NAC domain-containing proteins and TFs were most abundant in the up-regulated DEGs at 24 and 48 h of stress, while most of the down-regulated NAC DEGs were present at 48 h.

The AP2/ERF transcription factors are also involved in plant growth and development through interactions with cytokinins, gibberellins, and brassinosteroids [63]. Members of this family of TF also respond to abiotic stresses and interact with multiple hormones to effect stress responses [63,64]. For example, Arabidopsis plants overexpressing *AtERF15* were found to be more sensitive to ABA during germination and were more drought tolerant at the seedling stage [65]. In rice, *OsERF71* is involved in ABA signaling and proline biosynthesis in the drought tolerant upland rice variety *IRAT109*, and also confers increased drought tolerance when overexpressed in *Nipponbare* [66]. Wheat *TaERF3*-overexpressing lines accumulated more proline and chlorophyll, had reduced stomatal conductance and ROS accumulation, and were more tolerant to drought and salinity [67]. Some members of the AP2/ERF family, the dehydration responsive element-binding (DREB)-type TFs, act in an ABA-independent signaling pathway. In response to heat and dehydration, *DREB2A* was induced in Arabidopsis [68]. Arabidopsis plants overexpressing *DREB2A*, modified to be constitutively active, showed an improved performance under heat and drought stress conditions [69]. Transgenic rice expressing *OsDREB2A* regulated by the stress inducible promoter, 4ABRC, showed greater tolerance to severe drought and salt stress [70]. Expression of *DREB1A* under the control of a stress inducible promoter conferred drought, salt, and freezing tolerance in Arabidopsis [71], and drought tolerance in tall fescue [72]. In our study, DEGs encoding AP2/ERF TFs were more prevalent in the up-regulated DEGs, which were distributed ubiquitously across all time points, while the down-regulated DEGs were more prevalent at the 48 h time point. Most of the AP2/ERF DEGs annotated as DREB TFs were down-regulated at the later time points.

Other classes of transcription factors that were found to be less prominent in the transcriptome included GRAS (gibberellic acid insensitive (GAI), a repressor of the GA1-3 mutant (RGA), scarecrow (SCR)), WRKY, MADS box (Minichromosome maintenance factor 1, Agamous, Deficiens, Serum response factor), GATA, and heat shock transcription factors. GRAS proteins are involved in root and shoot development, axillary meristem development, phytochrome signaling, light signaling, and gibberellin signaling [73]. A rice *OsGRAS23* was found to be up-regulated in response to JA, salinity, and drought stress, and when overexpressed in rice conferred oxidative stress tolerance and improved drought resistance [74]. The GRAS domain-containing proteins were almost equally distributed between the up- and down-regulated DEGs, with the up-regulated DEGs being more prevalent at 24 h. WRKY transcription factors were previously shown to be involved in heat and drought stress responses [75,76]. When genes encoding a rice and wheat WRKY transcription factor were overexpressed in rice and Arabidopsis, respectively, enhanced heat/drought tolerance was observed [77,78]. The WRKY transcription factor DEGs were more prevalent in the up-regulated data set and were present across all time points. Members of the MADS-box protein family have been shown to be involved in transitioning to flowering, floral development, and patterning [79,80], and in drought, temperature, and oxidative stress responses [81]. The DEGs encoding MADS-box transcription factors were distributed almost evenly between up- and down-regulated DEGs and were more prevalent at the later stages of heat/drought stress. Plant GATA motifs and transcription factors were first discovered for their role in regulating nitrate assimilation [82,83] and light-responsive genes [84]. In rice studies, GATA transcription factors had elevated levels of expression in a salinity-tolerant variety when compared to a salinity-sensitive variety under control conditions. Furthermore, GATA transcripts were increased upon exposure to salinity, drought, and ABA in the sensitive variety, supporting the role of members of the GATA transcription factor family in mediating abiotic stress responses [85]. While there were only a few DEGs from this family, it is notable that they were almost all down-regulated and prevalent at the later stress time points. On the opposite spectrum, heat stress transcription factors (HSF) DEGs were only present in the up-regulated DEGs, and they were equally distributed across all time points. The HSFs are induced by multiple stresses such as heat, high light, and hydrogen peroxide, and they induce the production of heat shock proteins [86,87]. They may also have a role in sensing and regulating responses to ROS, which are often a component of abiotic and biotic stresses [88] and very likely a component of heat/drought stresses.

### 2.5. Analysis of Hormone-Related Genes Differentially Regulated during Heat/Drought Stress

**Figure 6:**
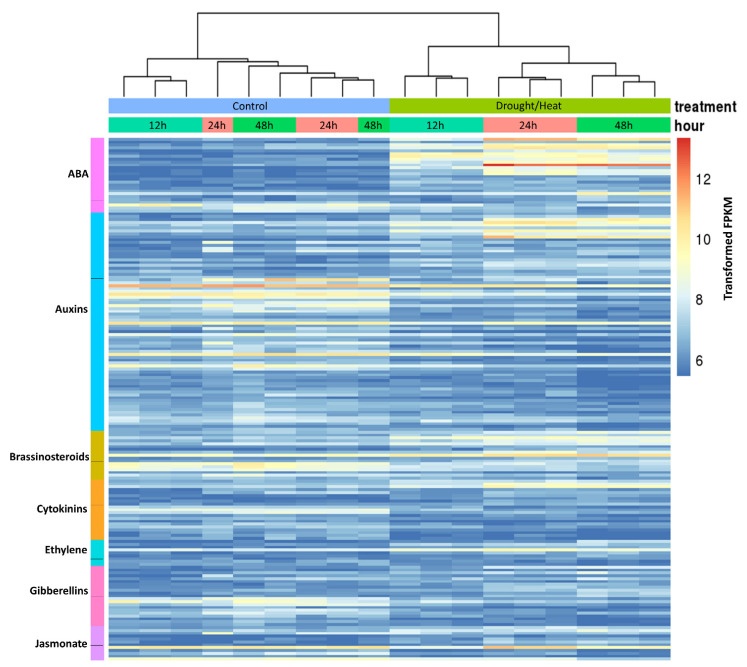
Heatmap of the 185 DEGs annotated as hormone related proteins that passed the differential expression filter of p-value 0.01, FDR 0.01, and log_2_-fold changes less than −2 or greater than 2 in at least one time point. Values were transformed with variance stabilizing transformation. Gradient bar on the right side of the heat map represents the transformed value of fragments per kilobase per million reads (FPKM). Treatments (control and heat/drought) and treatment times of 12, 24 and 48 h are as shown. Gene groupings of hormone related DEGs are shown on the left axis. Horizontal lines on the left-axis separate the up- and down-regulated DEGs within each class. Samples (columns) are clustered using Euclidean distance and the dendrogram is shown at the top of the figure. For the specific expression data for hormone related DEGs represented in the heatmap, see Supplementary Table S3.

Hormones are known to play an important role in the responses to abiotic stresses in plants. The up- and down-regulated DEGs were searched for DEGs encoding hormone-related proteins including those involved in biosynthesis, catabolism, modification, transport, or those acting as response factors for specific hormones. These DEGs encoding proteins involved in ABA, auxin, brassinosteroid, cytokinin, ethylene, gibberellin and jasmonate-related processes were used to create a heat map (Figure 6). Expression data for DEGs included in the hormone heat map are presented in Supplementary Table S3. Similar to the heatmap generated for TF (Figure 5), it should be noted that the 12 h control and stress treated plants are in a different clade from the 24 and 48 h control and treated plants, respectively. This could be related to the timing of the collection of the 12 h stress treatment occurring at the end of the light cycle and the 24 and 48 h treatments being taken early in the light cycle.

Auxins and cytokinins are important in the regulation of cell division in roots and shoots during normal plant growth and development and for modulating growth during stress responses. For the hormone auxin, many of the DEGs encoding auxin response factors (ARFs) and proteins regulated by the ARFs, auxin responsive proteins, were down-regulated and they were more prevalent at the later time points. The up-regulated DEGs encoding ARFs and auxin responsive proteins were distributed across all three time points. This is similar to what was reported in *Brachypodium*; members of the BdARF gene family were differentially regulated in response to hormone treatments and osmotic stress (PEG or NaCl treatments), with some being up-regulated and some down-regulated [89]. In *Brachypodium*, some *BdARF*s were up- and down-regulated differentially in root and leaf samples in response to abiotic stresses. However, in response to heat stress, all *BdARF*s were down-regulated except *BdARF4*, which was upregulated [89]. Another important consideration when looking at hormone responses in plants is their spatial distribution within the plant. Auxin efflux carriers can mediate auxin transport out of the cell and facilitate polar auxin transport to change the local concentrations of auxin during plant development and in response to environmental cues [90,91,92]. Some auxin transporters mediate auxin transport into or out of the ER lumen [93,94,95] and are thought to regulate auxin content at the subcellular level [96]. In our studies, the DEGs identified as coding for auxin efflux carrier components included some that were down-regulated at all three time points, but many were down-regulated only at the later time points. Only a few DEGs encoding auxin efflux carriers were found to be up-regulated.

There were also several DEGs identified as auxin import carriers and auxin transporter-like proteins that were down-regulated at the later stages of stress. It appears that many of the auxin transport genes were down-regulated in response to heat/drought stress.

As it is often the balance between auxins and cytokinins that is important in cell division and modulating growth, we also investigated genes encoding cytokinin-related proteins. The level of active cytokinins can be modulated through biosynthesis, modifications, and/or metabolism. There were several DEGs encoding cytokinin riboside 5′-monophosphate phosphoribohydrolases that are involved in cytokinin biosynthesis [97]. These DEGs were present mostly at the later stages of stress and were more often up-regulated. Interestingly, DEGs encoding purine permeases, which are thought to be involved in the transport of cytokinins across the plasma membrane, were more predominant at the later stages in the down-regulated DEGs [98]. Differentially expressed genes encoding cytokinin oxidases and N-glycosyltransferases, which function to inactivate cytokinins, were more often down-regulated and were present at all time points. Therefore, it seems likely that the level of cytokinins may increase overall due to a reduced breakdown and increased biosynthesis, while auxin-related activity may be more affected by a reduction in efflux carriers and auxin-response or responsive proteins at the later stages of heat/drought stress.

Abscisic acid (ABA) plays a key role during the plants’ response to many environmental stresses. Interestingly, for the DEGs related to ABA, many encoded proteins involved in ABA biosynthesis (9-cis-epoxycarotenoid dioxygenase) and catabolism (ABA 8′ hydroxylase) and both types of genes were up-regulated at all three time points, indicating that the levels of ABA are potentially being tightly regulated and/or differentially regulated in different tissues. There were also DEGs encoding GRAM (Glycosyltransferases, Rab-like GTPase Activators, Myotubularians) domain-containing proteins, many of which are ABA-responsive, that were highly up-regulated at all three time points. These proteins are thought to play a role in regulating environmental and hormonal signaling [99]. The few down-regulated ABA-related DEGs were annotated as proteins involved in ABA biosynthesis, an ABA receptor, and two ABA stress ripening proteins.

Gibberellins are involved in seed germination, stem elongation, root growth, leaf and fruit development, flowering, meristem maintenance, pollination, and abiotic stress responses [100]. The later steps in the GA biosynthetic pathway are catalyzed by cytochrome p450 monooxygenases, GA20- and GA3-oxidases, while GAs are inactivated by GA2-oxidases [101]. Gibberellins bind to GA GID1 (GA-Insensitive Dwarf 1) receptors, resulting in a conformational change that enables GID1 binding to DELLA proteins, which are then targeted for degradation, releasing their suppression of GA responses [102]. In response to abiotic stresses, GA activity is usually reduced, leading to plants with a reduced stature. Genes encoding enzymes that inactivate GAs, *GA2ox*s, are induced by various hormones and abiotic stresses including methyl jasmonate (*GA2ox3* and *GA2ox4*), ABA (*GA2ox6* and *GA2ox7*), osmotic, and salt stress (*GA2ox*) [103]. Decreasing active GA levels by overexpressing *GA2ox*s in tobacco and maize conferred an enhanced drought tolerance [104,105]. Interestingly, *GA2ox8*, which is expressed in stomata, is suppressed by ABA, and when overexpressed in Arabidopsis conferred drought tolerance [103]. The stabilization of Della proteins during salt stress leads to reduced growth and decreased ROS accumulation enabling the plant to survive under these conditions [106]. Gibberellins, together with ethylene, promote internode elongation in rice to escape submergence during flooding [107]. In Arabidopsis seeds exposed to high temperature, both the repression of GA biosynthetic genes and the induction of ABA biosynthesis are required to delay germination, suggesting that GA/ABA play a role in thermoinhibition [108]. Gibberellins play an important role in reducing growth in response to abiotic stresses. In response to heat/drought stress in *Lt*, DEGs encoding enzymes in the biosynthetic pathway (GA3- and GA20-oxidases; four up and four down), receptors (GID1; four up and three down), and inactivation pathway (GA2-ox-3 -5; three up and two down) were represented in the both the up- and down-regulated DEGs. Based on this, it appears that GAs are affected in the heat/drought response, but it is unclear what role they play. Additional tissue-specific expression studies would be required to determine where and how GAs are being regulated.

Ethylene is involved in many aspects of plant growth and development including seed dormancy and germination, flowering, fruit development, senescence, and abscission [109,110]. Ethylene also plays an important role in abiotic and biotic stress responses [111,112]. Ethylene is synthesized from S-adenosyl-L-methionine, which is converted to 1-aminocyclopropane-1-carboxylic acid (ACC) by ACC-synthase, and ACC is then converted to ethylene by ACC-oxidase [109,113]. Proteins involved in the perception of ethylene include members of the ethylene response (ETR), ethylene resistant sensor (ERS), and ethylene insensitive (EIN) gene families [110]. Transcription factors important in ethylene signaling belong to the ERF subfamily of AP2/ERF TF and include the dehydration responsive element binding protein (DREB) and ERF subclasses [114]. DREB2A is responsive to both drought and heat stress, and when a constitutively active DREB2A was over-expressed in Arabidopsis increased thermotolerance was observed [69]. Ethylene response factors ERF95 and ERF97 are involved in heat stress responses and overexpression of *ERF95* conferred enhanced basal thermotolerance in Arabidopsis [115]. In response to heat/drought stress in *Lt*, several DEGs encoding ethylene biosynthetic enzymes (four ACC oxidase and one ACC synthase) and ethylene receptors (EIN, ETR and ERS) were up-regulated. Several ERF TF, ERF-like 2c, and a DREB2A TF were up-regulated, and were more prevalent at the 24 and 48 h time points. A few ERF TF were also down-regulated. These results indicate a possible role for ethylene in the heat/drought response in *Lt*.

Brassinosteroids (BR) have been shown to be essential for normal plant growth and development. They are involved in seed germination, root and shoot growth, xylem differentiation, photomorphogenesis, and organ, flower, pollen, and fruit development [116]. Mutations in BR synthesis or signaling genes often lead to dwarf phenotypes and may lead to abnormal organ growth and development. Brassinosteroids interact with other hormones and transcription factors to control cell division and elongation, to regulate protein synthesis and gene expression, and to respond to abiotic and biotic stresses. Brassinosteroids are recognized by and bind to the cell surface receptors (BR insensitive 1, BRI1; BRI1-Like 1 and 3, BRL1 and BRL3) [116].

In response to heat/drought in *Lt*, there were multiple transcripts annotated as brassins present in the up- and down-regulated DEGs. A DEG encoding bru1, which is regulated by BR and involved in cell elongation [117], was up-regulated at 12 h. Other DEGs encoding BRL1 and BRL3 receptor proteins were up-regulated mostly at the later time points. While BRI1, BRL1, and BRL3 are all BR receptors, they may have specific roles in plant growth and stress responses based on their tissue-specific expression. While BRI1 receptors are expressed ubiquitously in plant tissues, the BRL1 and BRL3 receptors are expressed in vascular tissues and in the quiescent center and meristematic tissues of the root [118,119]. Recently BRL3 was shown to interact with and phosphorylate Regulator of G-Protein Signaling (RGS1), which is involved in sugar sensing and ROS production [119]. Both *brl3* and *brl1* mutants showed increased ROS bursts compared to wt, suggesting they may work together to inhibit ROS production [120]. Plants overexpressing BRL3 accumulated sugars in their root cells, which act as an osmoprotectant and help to overcome the effects of severe drought [121]. Based on the up-regulation of *BRL1* and *BRL3* observed in this study and the recent literature [119], these BRL proteins could be involved in sugar sensing, ROS production, vascular development, and/or cell wall metabolism in response to heat/drought in *Lt*.

Jasmonates are probably better known for their role in biotic stress responses, but they also play an important role in plant development and in responding to abiotic stresses [122]. Jasmonates and/or 12-oxophytodienoic acid (OPDA) are involved in seed germination, seedling development, senescence, flower development and timing, root growth, stomatal development, cold acclimation, and anthocyanin production [122,123]. The synthesis of JA begins in the plastid with the conversion of fatty acids in the chloroplast membranes to OPDA catalyzed by lipoxygenases (LOX), allene oxide synthase (AOS), and allene oxide cyclase. In the final steps of JA synthesis, which occur in the peroxisomes, OPDA is reduced by OPDA reductase, followed by three cycles of β-oxidation [123]. The jasmonate signaling pathway includes interactions with various transcription factors (bHLH, MYC, MYB, AP2/ERF-domain, EIN3, EIL, YABs, NAC, GAI, RGA) and hormones (auxin, ethylene, GA, BR, and salicylic acid) to elicit responses [124,125].

In *Lt* exposed to heat/drought stress, there were several DEGs involved in JA biosynthesis including LOX, AOS, and OPDA that were up-regulated and more prevalent at 12- and 24 h heat/drought stress, suggesting an increase in JA in response to heat/drought stress. In Arabidopsis, jasmonates negatively affect leaf growth through a reduction in cell number and cell size, by regulating the cell cycle and DNA endoreduplication. The inhibition of mitotic activity was related to a reduced expression of *CYCB1* [126]. In response to heat/drought stress in *Lt*, there were also many cyclin-B genes that were down-regulated and more prevalent at the later time points, possibly related to hormone-induced cell cycle changes. Foliar applications of JA or MeJA in various crop species have been reported to improve plant performance under oxidative, metal, temperature, salinity, and drought stress conditions, partly through improved control of ROS (decreased production, increased removal) and interactions with other plant hormones and transcription factors [124,125,127].

### 2.6. Functional Analysis of DEGs

Heat/drought stress resulted in significant changes in expression in a wide array of functional categories (Table 2). DEGs encoding for various proteins associated with the cell membrane and cell wall organization were shown to be differentially regulated. High temperatures can change the fluidity of plant cell and organelle membranes, which can result in changes to integral membrane proteins such as transporters, receptor proteins, receptor kinases, and ion channels [128]. Transporters play an important role in maintaining cellular homeostasis under stress conditions. There were many types of transporters which were more prevalent in the up-regulated DEGs at the 12 and 24 h time points, and in the down-regulated DEGs at the 48 h time point. The ATP-binding cassette (ABC) transporters were the most predominant type of transporter differentially expressed, with over 40 up-regulated DEGs at each time point, and about 30 down-regulated DEGs at the 12 and 24 h time points, and 55 at the 48 h time point. This family of transporters is involved in transporting a wide array of compounds, including hormones (ABA, auxins), peptides, lipids, inositol hexakisphosphate, glutathione S-conjugates, secondary metabolites, heavy metals and mineral ions [129]. They are located in various membranes of the cell including the plasma membrane, vacuolar membrane, mitochondrial membrane, plastid membrane, and the vacuolar and peroxisomal membranes, and are involved in detoxification, stomatal function, vacuolar sequestration, lipid catabolism and redox-active cytosolic FE/S protein assembly [129].

While the ABC transporters use ATP as an energy source, the Major Facilitator Superfamily (MFS) transporters use electrochemical gradients to transport nutrients, sugars, nitrate, hormones (ABA, GA), amines, inositol, phosphate, niacin, folate, and peptides [130]. There were 25, 20, and 27 up-regulated and 7, 13, and 24 down-regulated MFS transporters or MFS-domain-containing protein encoding DEGs at 12, 24, and 48 h, respectively. Several of the MFS transporters up-regulated DEGs at the 12 h time point were related to anion transporters in the chloroplast, plastid glucose transporters, and inorganic phosphate, protein, or sugar transporters. At the later time points, the up-regulated DEGs encoding MFS transporters were related to polyol, xylosyl, inositol, sugar phosphate exchanger, and sugar carrier proteins. Other DEG encoding transporters included sugar and potassium transporters that may be important for sodium ion and osmotic regulation. Mechano-sensitive channels, which may play a role in sensing osmotic stress and membrane tension [131], were more abundant in the up-regulated DEGs.

There were also many up- and down-regulated receptor kinase and wall-associated kinase DEGs, which play an important role in sensing environmental signals and transducing signals to other parts of the cell [132,133]. Overall, there were 778 up-regulated and 739 down-regulated DEGs annotated as kinases, with both up- and down-regulated kinases increasing with ongoing stress.

Phosphatases, which work in concert with kinases to regulate signaling pathways, were more often up-regulated and more prevalent at the later time points. Within the phosphatase-related DEGs, the metal-dependent phosphatases (PPM) were highly represented (71 DEGs) with most of them being up-regulated (58 DEGs) and more prevalent at the later time periods. Protein phosphatase 2C-As in this class of phosphatases are involved in stress-related signaling pathways involving ABA, mitogen-activated protein kinases, proteosomal degradation, and/or autophagy (in yeast) [134,135,136].

Plants utilize the ubiquitin 26S proteosomal degradation pathway to remove damaged proteins from the cytoplasm and nucleus during abiotic stress [29]. Ubiquitin ligase and transferase enzymes were more pronounced in the up-regulated DEGs and at the later time points. While the 26S proteasome pathway works in the nucleus and cytoplasm, Clp, Deg/HtrA, and FtsH proteases function in the chloroplast and mitochondria to remove damaged proteins [137]. Clp and FtsH proteases were up-regulated in response to heat/drought stress in *Lt*. These proteins are essential to maintain cell viability.

Another mechanism for dealing with damaged or misfolded proteins during abiotic stress involves the production of chaperones [138]. Genes encoding chaperones including heat shock proteins (HSP), DnaJ proteins, and late embryogenisis abundant (LEA) proteins are often induced in response to abiotic stresses to deal with protein aggregates, misfolded proteins, and denatured proteins. Overexpression of various HSPs in Arabidopsis, rice and tobacco conferred increased tolerance, or in some cases increased sensitivity, to abiotic stresses [138]. Dehydrins are present in the developing embryo and also accumulate in plants exposed to salinity and low temperature stress. They are thought to function as chaperones, providing membrane stability during stress responses, but also to bind metals and ROS to reduce oxidative damage [139]. These chaperone-related DEGs were predominant in the up-regulated DEGs at all time points.

Changes in the physical properties of the cell wall often occur when plants are exposed to environmental stresses. Cell walls are composed mainly of polysaccharides, lignin, proteins, and water. Modifications to these components can disrupt the structural integrity of the cell and alter cell growth and expansion. The main polysaccharides in the plant cell wall include cellulose, pectin, and hemicellulose. Many enzymes involved in cell wall modifications were differentially expressed in response to drought/heat stress. In general, they were more often down-regulated and present at the later time points. These included DEGs encoding cellulose synthase, laccase, xyloglucan endotransglucosylase, expansin, and pectin methy-, ethyl-, and acetyl-esterases. It has been well established that heat and drought stress can lead to reduced photosynthesis, thus limiting resources available for plant growth and development. Considering that cellulose synthesis is a large sink for carbohydrates, the reduction in photosynthesis, especially during the later stages of heat/drought stress, could lead to the reduction in cellulose synthesis [140]. Lignin is another important component of cell walls. Laccases oxidize the monolignal precursors leading to lignin polymerization, which strengthens the cell wall. Previous studies have shown a reduction in lignin formation in transgenic plants expressing cell wall modifying enzymes [141] and in bioenergy feedstocks harvested under drought conditions [142]. Laccase DEGs were found to be more numerous in the down-regulated DEGs at later stages of heat/drought stress. Chitinase was previously shown to be induced in response to dehydration in tall fescue [143], osmotic stress in tomatoes [144], and in response to heat stress in beans [145]. Chitinase and chitinase-like encoding DEGs were more prevalent in the up-regulated DEGs. Chitinase-like proteins have been shown to bind to glucan polymers and are thought to help regulate cellulose assembly [146]. The prevalence of these DEGs suggests maintaining the plasticity of the cell wall could be very important in surviving heat/drought stress.

The chloroplast houses the machinery for photosynthesis, but it is also important for synthesis of chlorophyll, hormones, lipids, fatty acids, carotenoids, and vitamins [147]. High temperatures and drought can lead to lipid peroxidation, increased ROS production and damage to chlorophyll, plastid membranes and photosystems necessary for photosynthesis [148,149]. In the current study, chloroplast-related DEGs were highly represented in both the up- and down-regulated DEGs. DEGs encoding for various components related to photosynthesis were found to be more predominant in the up-regulated transcripts at 12 h, but by 48 h they were more often down-regulated. Transcripts annotated as chlorophyll a-b binding proteins, which are part of the light-harvesting complex, were more prevalent at the later stages of stress and more often down-regulated, while those present after 12 h of stress were more often found to be up-regulated. Photosystem II-related up-regulated DEGs at 12 h of stress included transcripts encoding for the PSII protein D1, which needs to be synthesized during repair of PSII. The down-regulated DEGs at 48 h encoded psbP and several PSII 10 KDa proteins, which are thought to be involved in maintaining the oxygen-evolving complex (OEC) in PSII and may assist in folding and assembling thylakoid membrane complexes [150]. The reduced expression of *psbO*, also a part of the OEC, was reported in response to drought stress in barley [151]. Ribulose bisphosphate carboxylase (Rubisco) and Rubisco activase are key enzymes for photosynthesis. Protein stability and expression of genes encoding these proteins are both sensitive to temperature [152]. DEGs annotated as Rubisco or Rubisco activase were much more prevalent in down-regulated transcripts at the later time points. The decreased levels of Rubisco, combined with reduced expression of genes encoding essential components of photosystems suggest that the plant may be entering a survival state. It is interesting to note that there are a large number of down-regulated DEGs encoding cyclin-related proteins and ribosomal proteins that are more prevalent at 24 and 48 h time points.

Reactive oxygen species can serve as signaling molecules early in stress responses, but when present at elevated levels, they can cause cell death. DEGs were searched for transcripts encoding proteins (superoxide dismutases, catalases, peroxidases, cytochrome P450, ascorbate, glutathione, thioredoxin) that are involved in dealing with excess ROS in response to abiotic stress [153]. Interestingly, there were four DEGs encoding superoxide dismutases (one up-regulated at 48 h and three down-regulated at later time points), and only one up-regulated DEG encoding catalase present at 12 h, suggesting that these proteins may not be dominant in controlling ROS at these stages of the heat/drought stress response and/or that their activity may be controlled post-transcriptionally. It is interesting to note that in two citrus genotypes, elevated SOD, APX and CAT were found to be associated with the increased tolerance of Carrizo citrus to combined heat/drought stress [153]. Seven DEGs annotated as ascorbate peroxidase, which is thought to help protect photosynthetic machinery from oxidative stress during heat/drought stress combinations [154], were present in the up-regulated transcripts. Other ascorbate-related genes, such as reductases and oxidases, impact the level of ascorbate, which acts as an important antioxidant within the cell. DEGs annotated ascorbate reductases were more often up-regulated, while those encoding ascorbate oxidases were often down-regulated. DEGs encoding thioredoxin and glutathione-S-transferase were more prevalent in the up-regulated transcripts and increased with time, suggesting they may be important for controlling ROS as the heat/drought stress increased in *Lt* plants. Cytochrome P450s (CYPs) play important roles in biosynthesis of hormones, antioxidants, secondary metabolites, and in xenobiotic metabolism [155]. While there were many DEGs annotated as CYPs, they were fairly evenly distributed in the up- and down-regulated DEGs. Within the CYP encoding DEGs, there were transcripts encoding 20 members of the CYP71 subfamily that were up-regulated and eight members down-regulated, and ten DEGs encoding members of CYP86 subfamily that were down-regulated and two that were up-regulated. Members of the CYP71 subfamily were also highly represented in perennial ryegrass and tall fescue in response to heat stress, but in that case, they were more frequently down-regulated [156]. A member of CYP71 was also up-regulated in response to combined heat/drought stress in sorghum [157]. This data indicates that CYPs may be involved in a variety of the metabolic processes as the *Lt* plant copes with heat/drought stress.

### 2.7. Validation of RNA-Seq with qRT-PCR

**Figure 7:**
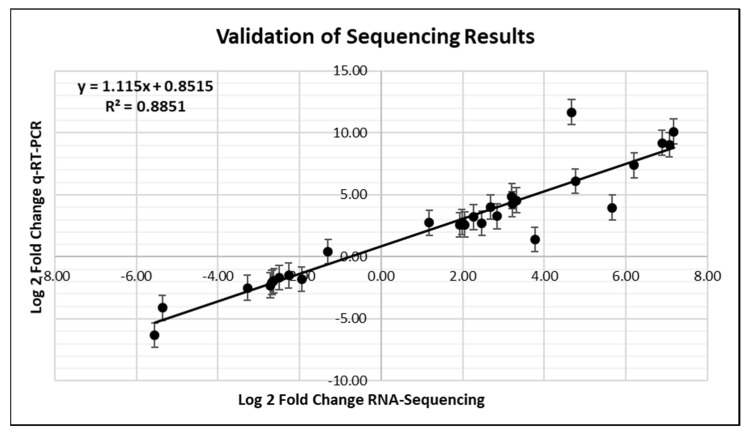
qRT-PCR validation of the RNA-Seq results of *Lolium temulentum* in response to drought/heat. Correlation between the relative quantification between control and heat/drought treated samples at 12, 24, and 48 h time points obtained through RNA-Seq analysis (log_2_ fold changes) and qRT-PCR analysis (log_2_ fold changes with standard error bars). Regression line and correlation coefficient are shown on the graph. Data are presented in Supplementary Table S4.

Ten DEGs (four down-regulated and six up-regulated) were selected for RNA-Seq validation to give a range of values across the different time points. The log_2_ fold changes in expression obtained by RNA-Seq and qRT-PCR for the selected DEGs are provided in Supplementary Table S4 and a graph of the comparisons is shown in Figure 7. Overall, the results showed general agreement between the two methods.

### 2.8. Comparison to Heat/Drought Studies in Other Species

Initial studies in the dicot species of tobacco and arabidopsis on combined drought and heat stress found that they shared DEGs in common with those observed in the individual heat or drought stresses; however, it also revealed many DEGs that were unique to the combined stress [10,11]. Common components of the drought/heat stress response generally include signaling proteins and molecules, ROS detoxification enzymes, antioxidants, chaperones, photosynthesis-related genes, and HSP [10,11,158,159]. In Arabidopsis, it was found that proline accumulated in response to drought stress, but surprisingly it did not accumulate in response to drought/heat stress [11]. In this study, similar to the findings in Arabidopsis, the proline biosynthetic genes were not differentially regulated in response to the combined stress. In contrast, proline was shown to accumulate in response to heat/drought stress in soybean [158]. The various classes of transcription factors differentially regulated in response to heat/drought stress in *Lt* were similar to those found in response to heat and/or drought stress in soybean, Arabidopsis, and wheat [158,159,160]. Genes encoding proteins involved in the degradation of chloroplastic proteins were found to be up-regulated in Arabidopsis [159], similar to what we observed in *Lt*. While the current studies show that there are many similarities in drought/heat responses between different species, there are also species-specific responses. These results indicate that future studies in a wider variety of species will be valuable to gain a better understanding of this combined heat/drought stress response.

## 3. Materials and Methods

### 3.1. Plant Materials

Seeds of *Lt* were obtained from Dr. Lloyd Evans (CSIRO, Canberra Australia). Seeds were grown and plants were allowed to self-pollinate for three generations. Seeds produced from the last generation were used to produce large quantities of seed for abiotic stress studies.

### 3.2. Growth of Lt for Experiments

**Figure 8:**
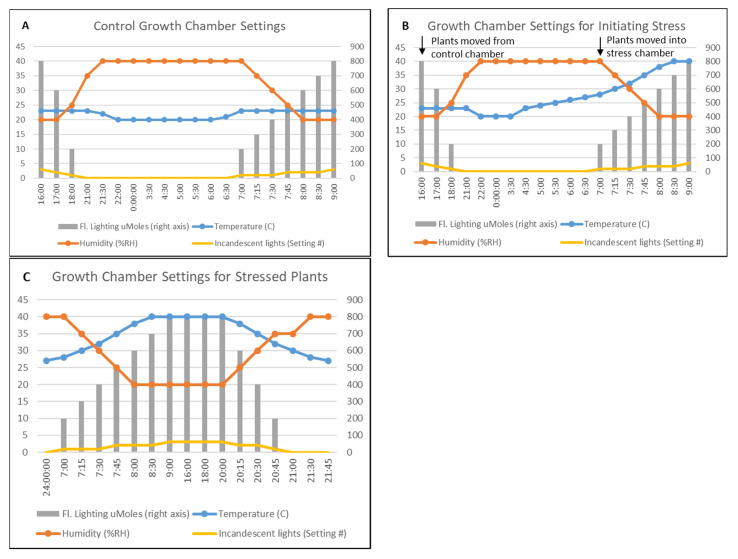
Growth chamber settings for experiments. The left axis numbers denote values for temperature in °C and the percent relative humidity (%RH). The right axis represent fluorescent lighting values in µMoles. Time of day is listed on the x-axis. (**A**) Control growth chamber settings, (**B**) Stress-initiation growth chamber settings. (**C**) Stress chamber settings.

Seeds (five/pot) of *Lt* L. cv. Ceres were planted in 10.2 cm tall × 8.8 cm square pots (TSD4, McConkey Co., Sumner, WA, USA) containing Sungro Professional mix MM840 PC RSi (Agawan, MA, USA). Plants were grown in Conviron PGR14 or PGR15 chambers set to 14-h day (7 AM–9 PM) and day/night temperatures of 23 °C/18 °C (Figure 8A) for 4–5 weeks until plants reached the 3-tiller stage. Plants were fertilized once/week with Technigro 20-18-20 all-purpose fertilizer (Sun Gro Horticulture, Hubbard, OR, USA).

### 3.3. Plant Treatments

Plants for the heat/drought stress were watered at 12 pm. Plants were transferred to the pre-stress Conviron PGR14 growth chamber with settings detailed in Figure 8B at 4 pm. The settings in this chamber were similar to the settings of the control chamber until 4:30 AM, when the temperature was set to increase 1 °C per 30 min until 7 AM. At 6:50 am the following morning, the plants were transferred from the pre-stress chamber to the stress chamber, where they remained for the rest of the experiment. Details of the settings for the stress chamber are shown in Figure 8C. Control plants remained in the original chamber and were watered at noon each day. Control and drought/heat treated samples (leaf tissues and root crown) were collected during the light cycle at 12 h (8 PM), 24 h (8 AM), and 48 h (8 AM). For each time point, the aerial portions of the plant and root crown were collected from three biological replicates for the control and heat/drought treated plants, placed in foil packets, quickly submerged in liquid nitrogen and stored at −80 °C until processing. Moreover, at each time point, two pots of the drought/heat treated plants were transferred back to the control chamber and watered to see if plants were able to recover. All plants from the 12, 24, and 48 h time points recovered, but at 72 h post-treatment, the plants were no longer viable, therefore this sample was discontinued.

### 3.4. Water Content Determination

Three leaves from each of the five plants/pot were removed and weighed to obtain the fresh weight (FW). The leaves were place in pre-weighed aluminum foil packets, dried at 80 °C for at least three days, and then weighed again to obtain the dry weight (DW). The water content was calculated using the formula ((FW − DW)/FW) × 100 = % WC.

### 3.5. RNA Sample Preparation and Illumina Sequencing

Total RNA was isolated from replicate control and heat/drought-treated samples using Trizol (Invitrogen, Carlsbad, CA, USA) following the manufacturer’s instructions. Samples were run on an agarose gel and RNA was measured on a DeNovix DS-11 spectrophotometer (DeNovix Inc., Wilmington, DE, USA) to assess the quality and concentration of the RNA. Samples were treated with DNase from the Turbo DNA-free kit (Ambion, Austin, TX, USA) and then purified using the RNA Clean and Concentrate kit (Zymo Research, Irvine, CA, USA) following the manufacturer’s instructions. The RNA samples were submitted to Oregon State University Center for Genome Research and Computing for library preparation and sequencing. The samples from three replicates of heat/drought-treated and control plants for each of the three time points were prepared for Illumina sequencing using the Wafergen RNA prep kit. Eighteen samples were sequenced on the Center for Genome Research and Biocomputing’s (CGRB) Illumina HiSeq 3000 with 100bp paired end.

### 3.6. Transcriptome Alignment and Analysis

The samples were trimmed to remove Illumina adapters and to remove low quality regions using Cutadapt with quality settings -q 15, 10 [161]. Reads were aligned to the *Lolium* reference transcriptome [16,17] with HISAT2 [162]. Sorting and processing of BAM files was completed with SAMtools [163]. Transcripts were quantified with StringTie [164,165], differential expression of expressed transcripts was performed with DESeq2 in R [166,167], and visualization of RNAseq differential expression results was completed with UpsetR [168] and Pheatmap [169].

Genes were annotated to a subset of the UniProt TrEMBL database [170] that included genes from grass species (Poaceae, Uniprot subset: https://www.uniprot.org/taxonomy/4479; accessed 1 February 2020). Genes were aligned to UniProt using BLASTX [171]. Gene ontology (GO) [172,173] identifiers were assigned using the UniProt alignments. GO term category upregulated or downregulated expression level differences were visualized using WEGO 2.0 [174], where significant differences were identified using a Chi-Square test for each GO category.

### 3.7. Validation of RNA-Seq with qRT-PCR

Validation of RNA-Seq results was assessed by comparing log_2_-fold change values of differentially up and down-regulated genes from RNA-Sequencing to those obtained using qRT-PCR. Primers from previous *Lt* stress studies were utilized for several transcripts that were differentially regulated in these studies. Additional transcripts were chosen based on their differential expression at multiple time points in this study. Primers for the new transcripts were designed using Primer3Web (v. 4.1.0). Only primers with single melt curves and within 90–110% efficiency were utilized for qRT-PCR analysis. A list of DEGs evaluated by qRT-PCR, their trinity numbers, and primer sequences are listed in Supplementary Table S4. Primer evaluation, cDNA preparation, reaction mixture and conditions, and qRT-PCR data analysis were performed as previously described [161]. Sample expression was normalized using the reference gene eukaryotic elongation factor 1-α [175].

## 4. Conclusions

Grasses are found growing ubiquitously in almost every environment in the world, but most commercially important grasses are grown and utilized in temperate climates. However, even in normally temperate environments plants are being exposed to more frequent extreme climatic events. In order to understand how grasses cope with concurrent heat and drought stress, it is important to identify not only what molecular processes the grasses mobilized in response to the stress, but also when these processes are activated over the course of the stress. The transcriptome analysis of the model grass *Lt* exposed to drought plus heat revealed a strong emphasis on transcriptional regulation, protein folding, cell cycle, cell parts, organelles, binding, transport, signaling, oxidoreductase, and antioxidant activity. These data provide a valuable first step towards unraveling the temporal relationships and interconnections between the various molecular processes utilized by the grasses in order to survive simultaneous exposure to heat and drought stress. The knowledge gained of genes and pathways that grasses use to respond to the combination of heat/drought will be useful in developing approaches to generate multi-stress tolerant grasses.

## Figures and Tables

**Table 1:** Sequence reads (forward and reverse) and percent alignment.

Time		Replicate 1		Replicate 2		Replicate 3	
(h)	Treatment	Reads	Alignment	Reads	Alignment	Reads	Alignment
12	Control	25998862	89.37%	30688704	91.05%	26382324	91.25%
24	Control	21245916	84.90%	20972450	84.05%	30003370	85.26%
48	Control	20772810	87.64%	27429856	85.73%	24471554	87.80%
12	Drought/heat	27944440	85.51%	36902414	86.40%	25405614	84.39%
24	Drought/heat	29130700	84.56%	28372698	86.37%	25201308	84.37%
48	Drought/heat	27912088	83.78%	25779500	83.51%	21224266	84.53%

**Table 2:** Biological functional analysis of DEG data.

Differentially Expressed Genes *	Total UP	Total DOWN	12-h UP	12-h DOWN	24-h UP	24-h DOWN	48-h UP	48-h DOWN
Phosphatases	161	81	77	30	112	38	111	70
PPM type	58	13	27	3	44	2	46	8
Kinases	778	739	306	201	447	236	515	576
Calcium/Calmodulin/Calcineurin	44	32	20	13	31	25	32	26
Transferases	407	428	181	110	215	185	256	340
Transporters (Tr.)	219	220	108	100	127	119	149	173
ABC Tr.	87	84	43	29	42	28	47	55
MFS domain-containing Tr.	59	34	25	7	20	13	27	24
Peptide Tr.			3	12	4	23	9	29
Proteases	106	45	58	14	68	24	82	37
Clp	15	6	10	1	11	3	12	5
Ftsh	8	0	6	0	8	0	7	0
Protease domain-containing	46	11	24	3	27	0	35	9
Ubiquitin (U)-related	147	70	66	15	80	27	97	54
U-Transferase	36	12	24	1	26	6	25	11
U-Ligase	43	26	22	6	26	12	29	23
U-Conjugating	5	7	3	4	3	6	3	5
U-carboxyl-terminal hydrolase	23	9	13	4	6	5	15	6
Chaperones								
Dehydrins, LEA, DNAJ	56	11	37	3	43	7	43	7
HSP	109	9	81	5	97	1	99	5
Photosynthesis								
Chloroplast	267	285	133	51	141	111	159	218
Photosystem	36	22	30	3	9	9	9	15
Chlorophyll	53	91	28	12	14	50	21	80
Chlorophyll a-b binding protein	23	82	18	10	7	45	9	76
Rubisco	12	41	10	7	7	20	9	34
Cytochrome c biogenesis	222	52	110	18	123	23	144	34
Cytochrome c oxidase	14	0	11	0	10	0	13	0
Cytochrome P450	83	81	43	40	38	45	48	53
Polyphenol oxidase	2	9	1	9	1	9	1	9
Peroxidase (ascorbate)	39 (7)	82 (0)	20 (4)	25	28 (7)	40	22 (6)	72
Glutathione S-transferase	41	12	18	5	31	6	34	14
Thioredoxin (reductase)	42 (7)	11 (2)	20 (4)	0	26 (6)	4	30 (7)	10 (3)
Cyclin	6	45	5	19	4	27	5	42
Ribosome	4	63	3	10	4	52	4	46
Lipoxygenase	17	11	6	2	15	1	7	11
Lipase	31	39	11	10	19	18	23	39
Phospholipase	14	21	5	5	7	10	8	15
Phospholipid-transporting ATPase	25	5	5	4	19	1	20	2
Cell wall related								
Cellulose synthase	9	35	3	7	5	12	4	32
Xyloglucan endotransglucosylase	10	27	3	4	3	9	7	24
Expansin	14	35	7	4	9	3	8	33
Glucanase	13	28	6	9	7	8	9	24
Pectin esterase	9	44	4	15	5	25	5	41
Laccase	10	36	5	3	4	17	4	33
Chitinase	12	1	7	1	7	0	10	0

* DEGs based on values with false discovery rate and *p*-values < 0.01 and |log2 fold changes| > 2.

## Data Availability

Data presented in this study are available in the supplementary tables provided with the manuscript. All sequences created for this study have been deposited into the NCBI Short Read Archive database uncer BioProject PRJNA769074.

## Supplementary Materials

The following are available online at https://www.mdpi.com/article/10.3390/plants10112247/s1, Figure S1: (A) Forty most significant GO categories at 12 h of stress, (B) Forty most significant GO categories at 24 h of stress, (C) Forty most significant GO categories at 48 h of stress, Table S1: List of UP- and DOWN-regulated DEGs between drought-heat treated and control plants at different time points, Table S2: Data for Upset R Plots shown in Figure 3, Table S3: Data for transcription factor and hormone heat maps, Table S4: Values for RNA-Seq vs qRT-PCR comparisons and qRT-PCR primer information.
