# Supplementary figures and images for: Transcriptome Analysis of Lolium temulentum Exposed to a Combination of Drought and Heat Stress
Source: Plants (Basel). 2021 Oct 21;10(11):2247. doi: 10.3390/plants10112247 (PMC8621252; doi:10.3390/plants10112247)

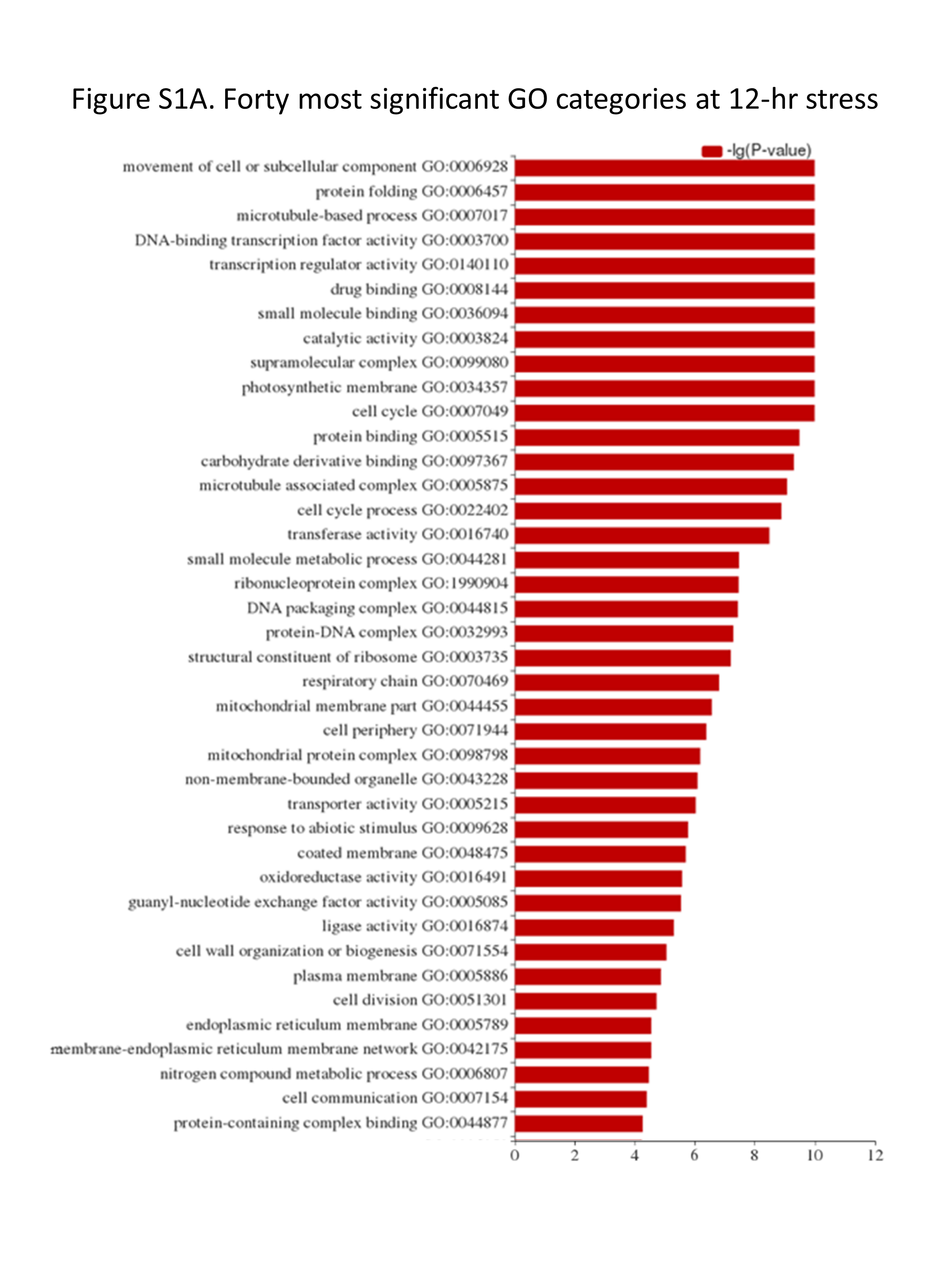

Supplement: Supplementary file 1 [file plants-10-02247-s001.zip › Figure S1A. P values top 40 Drought heat WEGO pics for p values.png]

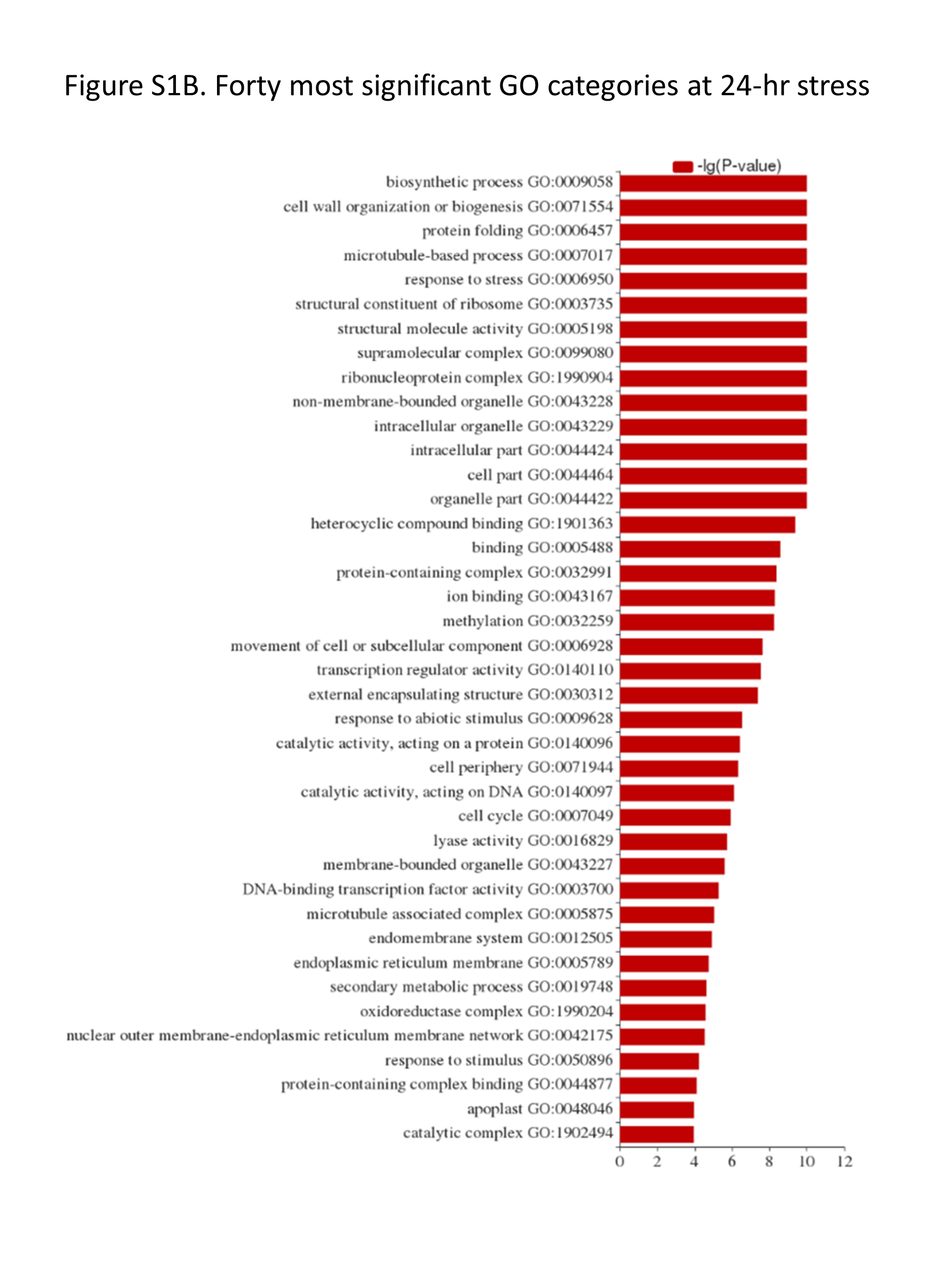

Supplement: Supplementary file 1 [file plants-10-02247-s001.zip › Figure S1B. P values top 40 Drought heat WEGO pics for p values.png]

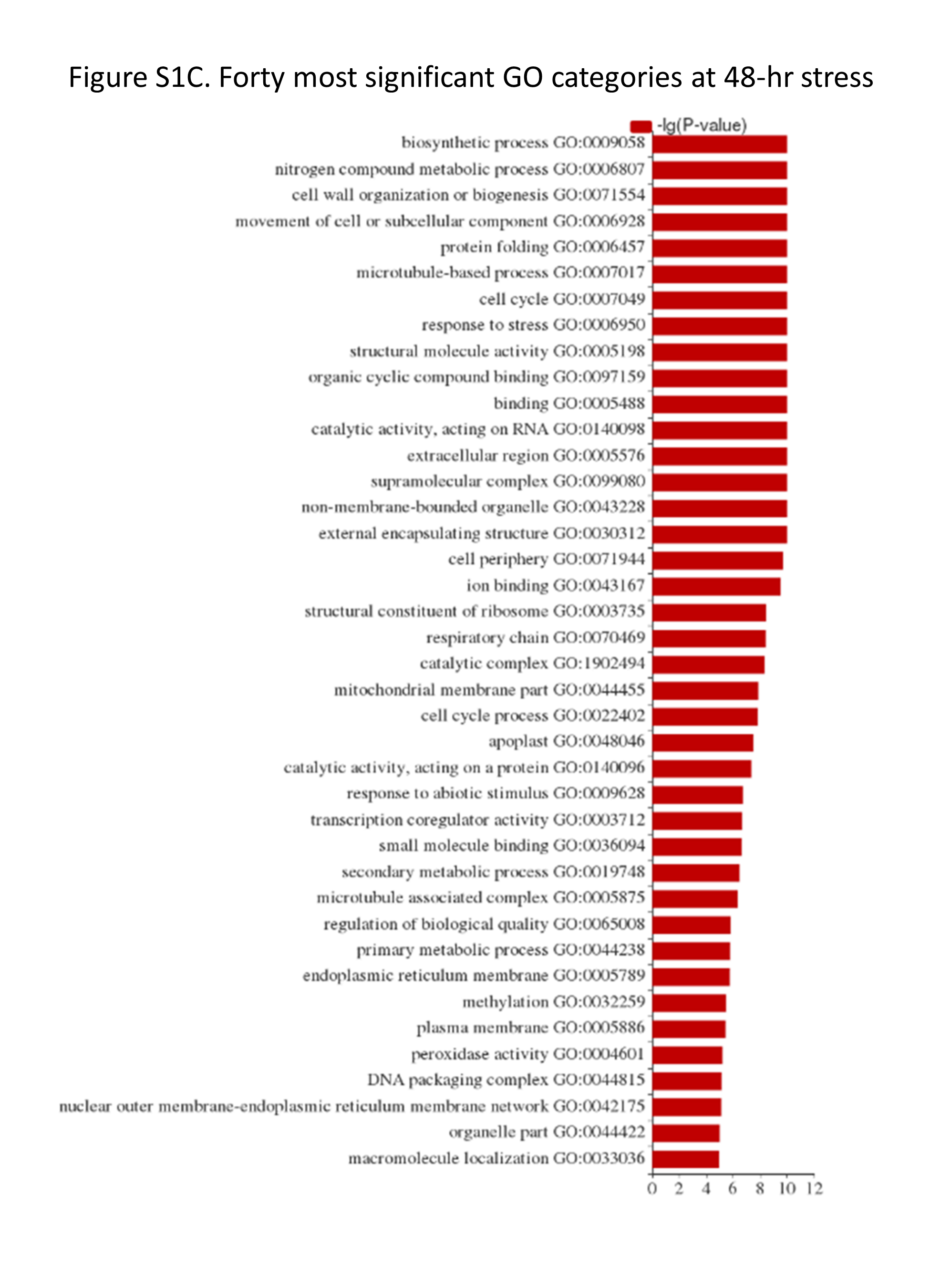

Supplement: Supplementary file 1 [file plants-10-02247-s001.zip › Figure S1C. P values top 40 Drought heat WEGO pics for p values.png]
